# Cor Triatriatum in a Pediatric Patient, Accidental Point of Care Ultrasound (POCUS) Discovery

**DOI:** 10.24908/pocusj.v10i01.17718

**Published:** 2025-04-15

**Authors:** Tareq Alhaddad, Amr Hamid, Abdulbaset Mohammed, Hana Mohsen

**Affiliations:** 1 Pediatric Emergency Department at Alzamalah hospital, Taiz City, YEM; 2 Faculity of Medicine and Health Science, Taiz University, Taiz City, YEM; 3 Pediatric and Emergency Department at Alzamalah hospital, Taiz City, YEM; 4 Internal Medicine Department, Alsafwa Hospital, Taiz City, YEM; 5 Faculty of Medicine, Sanaa University, Sanaa, YEM; 6 Pediatrics, Al Kuwait Hospital, Sanaa, YEM

**Keywords:** Cardiac POCUS, Cor Triatriatum, Emergency Ultrasound, Lung POCUS

## Abstract

Cor triatriatum is a rare congenital heart defect, occurring in less than 0.1% of all such cases. It is characterized by a fibrous membrane dividing the atrium into two compartments. While often asymptomatic in infants and children, it can cause symptoms like shortness of breath, fatigue, and malnutrition. Early diagnosis and treatment are crucial to prevent further cardiac complications and ensure normal childhood growth and development.

We discuss the case of a 4-year-old girl diagnosed with cor triatriatum, who was admitted to the emergency room exhibiting symptoms of pneumonia and poor weight gain. A point of care ultrasound (POCUS) test revealed a membrane in the left atrium. The child was admitted and treated for pneumonia and then referred for surgical repair of her heart defect. Following surgery, she was discharged home in stable condition. This case emphasizes the importance of POCUS — particularly in resource-limited settings — for early detection and treatment of congenital heart defects in children. It also shows the need for comprehensive assessment of symptoms for timely diagnosis and management of rare cardiac anomalies, such as cor triatriatum.

## Introduction

**Figure 1.**
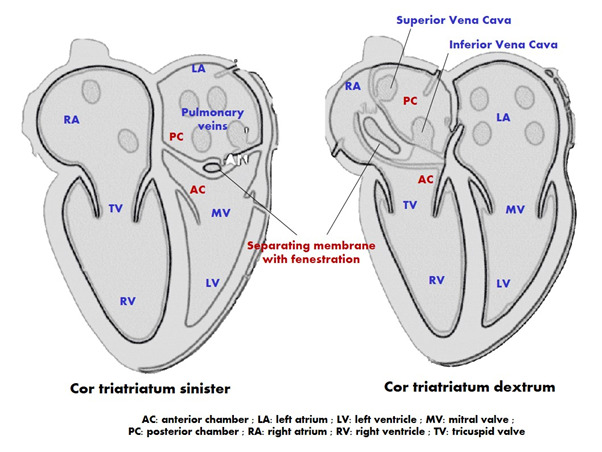
From: C. A. Minoiu, A. Meduri, B. Merlino, et al. (2015) Cor triatriatum: the role of cardiac-MR in establishing a correct diagnosis. ECR 2015 Poster C- 2316. DOI: 10.1594/ecr2015/C-2316. © European Society of Radiology.

Cor triatriatum is a rare congenital heart defect that makes up about 0.1% of all congenital heart diseases [1]. This condition is characterized by a thick, fibromuscular membrane that divides the atrium into two separate chambers (Figure 1). It typically affects the left atrium (cor triatriatum sinister) and only occasionally the right atrium (cor triatriatum dexter) [2]. The clinical symptoms primarily depend on factors such as the size of the membrane's orifice, the defect's shape, and the pressure difference between the two chambers [3].

This anomaly often goes unnoticed in infants and children as it typically presents no symptoms. However, as the child grows and becomes more active, they may start showing signs such as breathlessness, fatigue, and malnutrition. Cor triatriatum is characterized by a partition within the atrium, creating two chambers. In contrast, a supravalvular mitral ring is a fibrous membrane located above the mitral valve, which can obstruct blood flow but does not divide the atrium into chambers. Distinguishing between these involves imaging techniques, where cor triatriatum shows a clear division of the atrium, while a supravalvular ring appears as an obstruction at the valve level.

In our hospital, formal echocardiography is not available in the emergency department. However, point of care ultrasound (POCUS) is accessible and is routinely used by clinicians for initial cardiac and respiratory assessment.

Early detection and treatment of cor triatriatum are vital to prevent additional heart complications and ensure normal growth and development [4]. However, owing to its rarity and asymptomatic nature, cor triatriatum is commonly diagnosed later in childhood or even in adulthood [5]. We present a case of a 4-year-old girl who came to the emergency department with breathing difficulties accompanied by hypoxia and chronic malnutrition. POCUS was performed in this case to assess the patient's respiratory status and to rule out potential cardiac causes of her symptoms. POCUS identified the presence of a cardiac membrane that was obstructive, resulting in impaired blood flow. Additionally, there were no significant structural abnormalities noted, and the overall cardiac function appeared preserved. She was admitted for pneumonia treatment before being referred for surgical repair. This case report highlights a unique situation of recurrent chest infections and malnutrition in a child, leading to the unforeseen discovery of cor triatriatum using POCUS.

## Case Presentation

**Figure 2.**
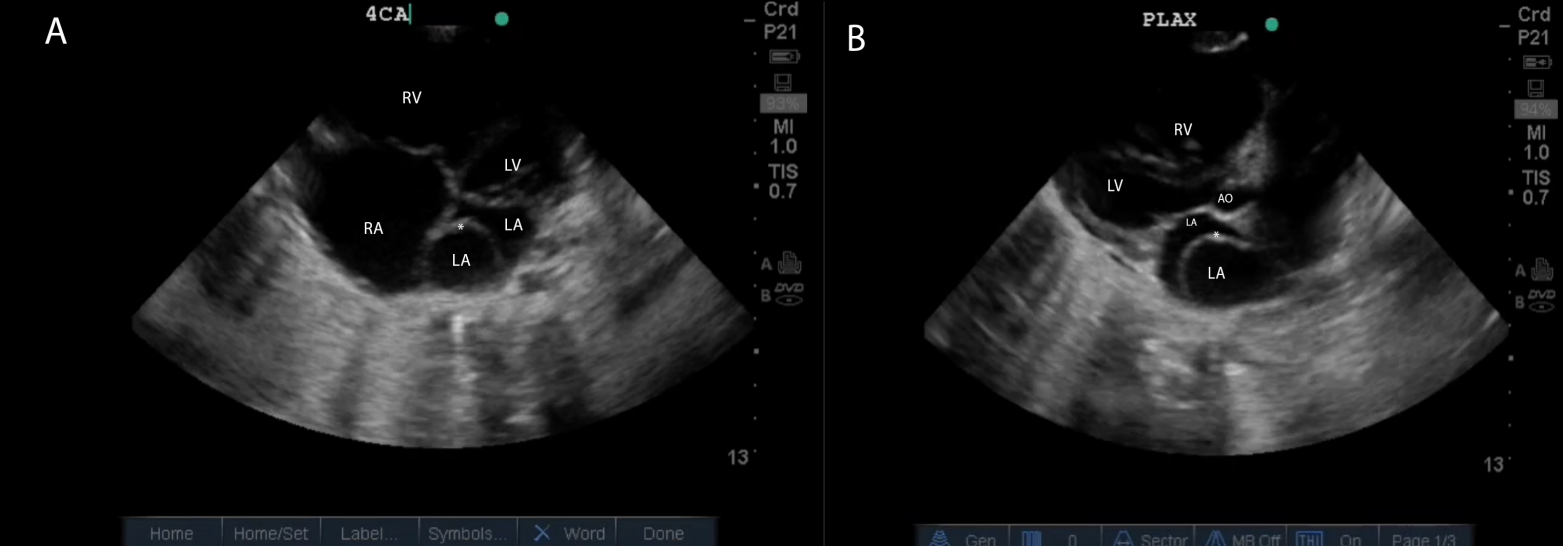
Cardiac point of care ultrasound (POCUS) showing Cor triatriatum. A. Apical four-chamber views (4CA) revealing a membrane. (White star) subdividing the left atrium into two chambers. B. Parasternal long axis view (PLAX), LA: left atrium; LV: left ventricle; RV: right ventricle; RA: right atrium; AO: Aortic root.

**Figure 3.**
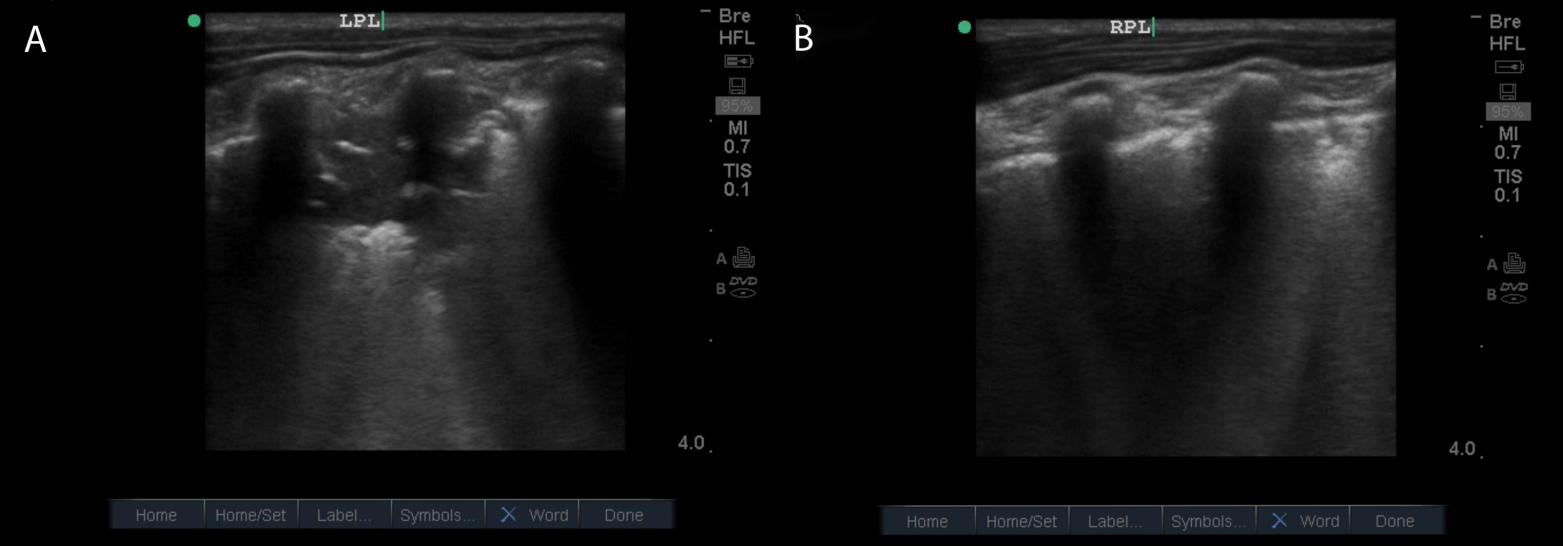
Lung point of care ultrasound (POCUS) showing pneumonia. A. Longitudinal views of the left posterior lung (LPL) showing large lobar consolidation. B. Longitudinal views of right posterior lung (RPL) showing subpleural consolidation with confluent B-lines.

A 4-year-old girl with a history of poor weight gain presented to the emergency department with respiratory distress and hypoxemia. She suffered from cough and fever for 7 days, and her oxygen saturation was 86% on room air. Per maternal report, she had been evaluated by many medical doctors previously and treated for pneumonia. One year prior to presentation, she was diagnosed with asthma and received salbutamol puffs and prescriptions containing prednisolone. Respiratory symptoms subsequently improved until about 2 months before the emergency room visit. At that time, she was diagnosed with an acute lower respiratory tract infection and started taking amoxicillin with no improvement; 10 days later, she developed cough with upper respiratory symptoms and fatigue. Her mother reported that she had multiple outpatient therapeutic feeding clinic visits and had not seen any improvements in nutrition or weight gain. On the day of presentation to the emergency department, her mother reported that her child was feeling tired and had poor feeding, as well as a fever and progressively worsening work of breathing over the course of 3 days, with 1 day of severe shortness of breath and cough. She had taken paracetamol earlier that day and had undergone repeated treatments with a salbutamol nebulizer prior to her arrival with no improvement. On presentation she was found to have marked tachypnea and respiratory distress, as well as hypoxia with an oxygen saturation level of 86% on room-air. Bilateral crepitation was noted on pulmonary auscultation, but no obvious heart murmurs were heard on cardiac auscultation. Laboratory tests revealed low levels of hemoglobin and a high white blood cell count. POCUS examination was performed to assess the patient's respiratory status. Surprisingly, POCUS revealed an abnormal septal membrane dividing the left atrium, consistent with cor triatriatum, as well as the presence of consolidative lung disease in the left and right posterior lobe, possibly due to recurrent chest infections (Figure 2, Figure 3). This unexpected finding prompted further investigation and subsequent management planning. The patient was admitted and started on intravenous ceftriaxone for 5 days and a course of high-calorie supplements to address the malnutrition. Over the course of the patient's hospitalization, auscultation showed improvement in aeration, oxygen saturation improved to 96%, and the work of breathing improved significantly. These improvements allowed her to be referred to a cardiologist for further management of the heart condition.

## Discussion

Cor triatriatum is a rare congenital heart defect characterized by the presence of a fibromuscular membrane that divides the atrium into two chambers. In non-obstructive cases, it is often asymptomatic in infants and young children and may not be diagnosed until later in life. However, as children get older and more active, symptoms such as shortness of breath, fatigue, and poor weight gain may appear. This condition can be potentially dangerous because it can impede normal blood flow across the mitral valve and restrict pulmonary venous return to the left heart.

The obstruction created by the membrane can lead to elevated pulmonary venous pressure, resulting in congestion in the pulmonary circulation. This increased pressure can cause symptoms of pulmonary congestion, such as tachypnea and respiratory distress. Over time, prolonged elevated pressures may lead to complications such as pulmonary hypertension and right heart failure, emphasizing the importance of early detection and management.

In this case, a 4-year-old girl presented with respiratory distress, hypoxia, and poor weight gain with a recurrent chest infection. Initial evaluation and treatment focused on lung infection, as these were the most common causes of her symptoms. However, despite previous interventions, her symptoms worsened, suggesting the need for further investigation. Clinical symptoms mainly depend on the size of the membrane and the morphology of the defect [6–8]. The assessment and diagnosis of cor triatriatum includes diagnostic and imaging studies such as chest X-rays, electrocardiograms, and echocardiography. Diagnosis is best made by echocardiography and/or cardiopulmonary POCUS. POCUS allows for real-time visualization of lung and cardiac structures and function, enabling clinicians to identify anatomical abnormalities [7]. In this scenario, POCUS provided immediate imaging evidence of the abnormal septal membrane, leading to the timely recognition of cor triatriatum. This early diagnosis allowed the initiation of appropriate treatment strategies, including consultation with a pediatric cardiologist and consideration of potential surgical intervention [11, 13]. The patient underwent corrective cardiac surgery and was discharged home in stable condition. Follow-up with her cardiac surgeon 4 months postoperatively indicated that she was stable and her symptoms had significantly improved, including a reduction in respiratory infections and marked weight gain.

It should be noted that a late diagnosis of cor triatriatum (as in this case) is not uncommon, as the disease is rare and often asymptomatic in young children. Atypical presentation with recurrent pulmonary infections and malnutrition symptoms may contribute to the late recognition of underlying cardiac abnormalities. Medical treatment for cor triatriatum depends on the symptoms [3]. The treatment of choice is surgical resection, which is generally well tolerated with a good prognosis. Long-term cardiovascular follow-up is needed to monitor for rare sequelae [14, 15].

## Conclusion

Cor triatriatum is a rare congenital cardiac anomaly. It is often asymptomatic in infants and children but can present with respiratory distress, fatigue, and poor weight gain as the child grows and becomes more active. In this case, a 4-year-old girl with a history of recurrent chest infections and poor weight gain presented with respiratory distress and hypoxia. POCUS played a vital role in the diagnosis, revealing the presence of a cardiac membrane indicative of cor triatriatum. The accidental detection of cor triatriatum in a pediatric patient with recurrent chest infections and malnutrition highlights the importance of considering underlying cardiac anomalies in cases with unexplained clinical presentations. POCUS serves as a valuable tool for prompt diagnosis and appropriate management, ultimately improving patient outcomes.







## Name of Department and Institution where the work was done

Alzamalah hospital
